# Neurochemical Profiles of Prefrontal Cortex and Hypothalamus at 3 and 7 T During Controlled Euglycemia: Evaluation in a Cohort With Type 1 Diabetes

**DOI:** 10.1002/nbm.70108

**Published:** 2025-07-27

**Authors:** Young Woo Park, Dinesh K. Deelchand, James M. Joers, Anjali Kumar, Alison Bunio Alvear, Amir Moheet, Elizabeth R. Seaquist, Gülin Öz

**Affiliations:** ^1^ Department of Radiology, Center for Magnetic Resonance Research University of Minnesota Minneapolis Minnesota USA; ^2^ Henry M. Jackson Foundation for the Advancement of Military Medicine Inc Bethesda Maryland USA; ^3^ Department of Radiology Uniformed Services University of the Health Sciences Bethesda Maryland USA; ^4^ Department of Medicine University of Minnesota Minneapolis Minnesota USA

## Abstract

With the increasing adoption of ultrahigh‐field MRI scanners, there is a growing interest in migrating MRS studies from 3 to 7 T. Prior field comparisons of MRS in healthy volunteers demonstrated better reliability of quantification at 7 T, particularly for weakly represented metabolites. Neurochemical quantification has not been compared at 3 T versus 7 T in clinical cohorts and under controlled physiological conditions. In this exploratory study, we analyzed MRS data from the hypothalamus and prefrontal cortex volumes of interest (VOIs) that were collected from the same individuals with Type 1 diabetes at 3 and 7 T as a part of a larger study investigating cerebral responses to glycemic changes. Seventeen individuals underwent MRS during euglycemic clamps at both 3 T and 7 T, allowing us to compare metabolite concentrations obtained at the two fields with a consensus‐recommended short‐echo semi‐LASER protocol under the same physiological conditions. Our aim was to examine whether there are systematic biases in neurochemical concentrations measured at 3 T versus 7 T and to assess whether creatine (tCr) ratios would reduce or eliminate such biases. High‐quality spectra were obtained from both VOIs and fields, with 8–15 reliably quantified (mean Cramér–Rao lower bounds ≤ 20%) metabolites from LCModel. Whereas neurochemical profiles were highly similar between 3 and 7 T, several metabolites exhibited systematic differences with water‐referenced quantifications, such as glucose + taurine (Glc + Tau) and phosphoethanolamine. Quantifications with tCr as reference did not alleviate the biases and, in fact, resulted in a larger number of significant differences due to systematic biases in the tCr concentration. Pearson correlation analysis showed significant associations for several metabolites between 3 and 7 T, suggesting that interindividual differences in neurochemical levels are detectable. Associations were stronger when using tCr ratios. Importantly, hypothalamic Glc + Tau showed a strong correlation between fields at these tightly regulated euglycemic conditions, opening the possibility to detect individualized glucose concentrations in this brain region that participates in the regulation of blood glucose levels.

## Graphical abstract

This study compares MRS data acquired at 3 T and 7 T from the hypothalamus and prefrontal cortex in individuals with Type 1 diabetes. Despite similar metabolite levels, systematic differences were observed, particularly in phosphoethanolamine and the sum of glucose and taurine. Using creatine ratios did not eliminate and, in fact, exacerbated these biases. Significant correlations between concentration estimates at 3 T and 7 T suggest that inter‐individual differences in select neurochemical levels are detectable over a narrow range of blood glucose levels.

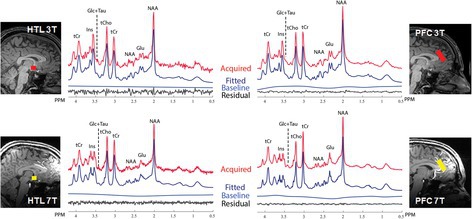

## 1 Introduction

In MR engineering, the now widely available 3 T systems are considered high‐field (HF), whereas ultrahigh‐field (UHF) designates field strengths ≥ 7 T [1]. In 2017, the FDA approved the first 7 T UHF MRI scanner for clinical use in the United States [2], paving the way for broader clinical applications. Since then, many leading research institutions and hospitals have adopted 7 T UHF MRI for studies in neuroscience and clinical research domains [3]. With the increasing adoption of the UHF scanners, there is growing interest in migrating existing 3 T MRS studies to 7 T [4, 5].

Previous comparisons of MRS data obtained from the same healthy individuals at HF and UHF have demonstrated superior signal‐to‐noise ratio (SNR) and spectral resolution at UHF, resulting in more reliable and reproducible quantification of metabolites such as gamma‐aminobutyric acid (GABA), glutamate (Glu), glutamine (Gln), and lactate (Lac) [6, 7, 8, 9]. Glucose (Glc) quantification, on the other hand, has been found to be more reliable at HF due to a simpler spectral pattern at 3 T versus 7 T [7, 8].

Neurochemical quantification has not been compared at HF versus UHF in clinical cohorts and under controlled physiological conditions, particularly with respect to glycemic levels. Namely, a key aspect that has been overlooked in prior field comparisons is the dynamic fluctuations of glucose levels throughout the day. Brain glucose levels are linearly associated with plasma glucose concentrations [10, 11], and plasma/brain glucose fluctuations may also affect other neurochemical levels [12, 13]. The variability of the plasma glucose level can be avoided via the glucose clamp technique [14], thereby stabilizing brain glucose levels and any other neurochemicals that are metabolically linked to cerebral glucose during the MRS measurement.

In this study, we used a subset of data from a study focused on measuring cerebral responses to glycemic changes in individuals with Type 1 diabetes (T1D) [15] before and after an intervention in the cohort with diabetes. Participants were scanned at both 3 and 7 T, with an interval of 3–29 months between sessions. Each scanning session included a period in which plasma glucose was stabilized between 90 and 100 mg/dL, prior to clamping the blood glucose at the predefined levels to address specific hypotheses. During the baseline euglycemic clamp, the participants were monitored and maintained at a target glucose of 95 ± 5 mg/dL, which is normal (euglycemic) for healthy individuals. MR spectroscopy (MRS) data were collected from the hypothalamus (HTL) and prefrontal cortex (PFC) volumes of interest (VOIs) at both field strengths, providing unique data from a clinical cohort under the same well‐controlled physiological state (euglycemia) at HF and UHF.

We aimed to investigate whether there are systematic biases in neurochemical concentrations obtained with an advanced, consensus‐recommended [16] MRS protocol on 3 T versus 7 T from two VOIs relevant to cognitive and metabolic investigations. We further aimed to determine whether quantification referencing to total creatine (tCr) reduces or eliminates such biases over referencing to water. Finally, we investigated associations between metabolite levels obtained at 3 and 7 T to determine if interindividual differences in neurochemical concentrations in this clinical cohort are detectable under tightly controlled physiology.

## 2 Methods

### 2.1 Participants and Study Design

Individuals with T1D were enrolled in this study following approval by the Institutional Review Board (IRB) at the University of Minnesota Medical School. *Inclusion criteria* included T1D (diagnosed on clinical or laboratory grounds), ages 18–65, hemoglobin A1c (A1c) < 8.5%, and diabetes duration of 2–25 years. *Exclusion criteria* included impaired awareness to hypoglycemia as determined by Clarke et al. [17] and Gold et al. [18] questionnaires, uncontrolled hypertension (blood pressure above 145/95 at screening), evidence of autonomic neuropathy (presence of orthostatic hypotension or history of gastroparesis), proliferative retinopathy, glomerular filtration rate (GFR) below 45, history of myocardial infarction, stroke, seizures, neurosurgical procedures, major depression requiring hospitalization within the last 5 years, current substance abuse, arrhythmias, and use of drugs that can alter glucose metabolism (other than insulin). All participants also met MRI safety requirements (weight less than 135 kg, the absence of metallic substances in their body). Seventeen participants were scanned at both 3 and 7 T (Table 1).

**TABLE 1:** Participant demographics and clinical characteristics. *P* value was computed with paired, two‐tailed *t*‐test.

Mean (±SD)	7 T	3 T	*p*
Age (year)	30.2 (±9.3)	30.6 (±9.3)	0.168
Sample size (male/female)	17 (8:9)		—
BMI	29.6 (±6.4)	29.8 (±6.4)	0.552
A1C (mg/dL)	7.1 (±0.8)	6.8 (±0.6)	0.214
T1D duration (year)	15.9 (±8.2)	15.7 (±7.1)	0.751
Systolic blood pressure (mmHg)	123.4 (±7.9)	123.1 (±9)	0.926
Diastolic blood pressure (mmHg)	76.6 (±7.5)	76.5 (±6.9)	0.957
First scan	9	8	—
Blood glucose during HTL MRS (mg/dL)	92.9 (±4)	99.9 (±9.7)	0.060
Blood glucose during PFC MRS (mg/dL)	95.9 (±6.5)	92 (±4.9)	0.054

Each participant underwent a clinical evaluation 1 week before the scan, whereby body mass index (BMI), A1c levels, duration of diabetes, and systolic and diastolic blood pressure were recorded. On the day of imaging, intravenous catheters were placed, and an intravenous infusion of insulin was started and adjusted as necessary to bring blood glucose to 95 mg/dL. Potassium phosphate was infused at 4 mEq/h as long as the insulin was being infused. Serum glucose was collected every 5 min for measurement of glucose on a nearby glucose analyzer system (Analox Instruments, Hammersmith, London, UK) to guide adjustments in the glucose infusion rate. The glucose clamp [14] began when the participant was at 95 mg/dL. The intravenous insulin infusion was fixed at 2.0 mU/kg/min, samples for blood glucose were collected every 5 min, and an intravenous infusion of glucose (20% dextrose) was administered as necessary to maintain blood glucose at 95 mg/dL in the scanner.

### 2.2 Acquisition of MR Data

Commercial 3 T and 7 T systems manufactured by Siemens Healthineers (Erlangen, Germany) were used for this study. We acquired 3 T data using a Siemens Prisma^Fit^ scanner (Syngo VE11C) with a 64‐channel receive head coil and KinetiCor (San Diego, CA, USA) motion tracking system [19]. We acquired 7 T data using a Siemens 7 T Plus scanner (Syngo VB17 and VE12U‐AP01) with the commercial 1‐channel transmit and 32‐channel receive head coil from Nova Medical (Wilmington, MA, USA), a Metria Innovation (Wauwatosa, WI, USA) motion tracking system [20], and a BaTiO_3_ pad [21] placed over the forehead to achieve sufficient B_1_^+^ in the PFC voxel.

T_1_‐weighted MPRAGE (TR/TI/TE = 2530/1200/3.65 ms at 3 T; TR/TI/TE = 2890/1500/2.42 ms at 7 T) structural images (1 × 1 × 1 mm^3^ resolution) were collected prior to MRS measurements for prescriptions of VOIs and tissue fraction estimations. For 7 T, AFI images [22] (3D GRE, TR/TE = 60/2.33 ms, Ref Amp. 220 V, 3 × 3 × 4 mm^3^ resolution) were also collected to compute the 90° transmit voltage value for each VOI.

Single‐voxel MRS data were collected from PFC (24 × 30 × 12 mm^3^) and HTL (13 × 12 × 10 mm^3^), regions believed to be involved in hypoglycemia awareness [23, 24] and the counterregulatory hormone responses to hypoglycemia [25]. MRS data were collected while participants were maintained at euglycemia, with a target glucose level of 95 mg/dL. We used a short‐echo (TR/TE = 5000/28 ms at 3 T; TR/TE = 5000/26 ms at 7 T) semi‐LASER protocol [15, 26, 27], with 64 transients (~6 min) for PFC and 256 transients (~22 min) for HTL. Prospective motion and shim correction was applied as described previously [15, 28] to ensure sufficient numbers of transients were collected with high spatial integrity for both regions. Two sets of water references were obtained: (1) without the VAPOR water suppression pulses [29, 30] and gradients for metabolite quantification and (2) with the VAPOR gradients enabled but no RF pulses for eddy‐current corrections. Four transients were collected for each water reference set, with half collected before and half after the metabolite acquisition.

### 2.3 Analysis of MRS Data

MRS data were preprocessed (single‐shot phase and frequency alignment and eddy‐current correction) with MRspa [31] prior to quantification with LCModel [32] (Version 6.3.1‐N). Basis sets for 3 and 7 T were each separately simulated via density‐matrix formalism using known chemical shifts and J‐coupling constants [33] and the same RF pulse shapes, duration, and inter‐pulse delays as used in the sLASER sequence for measurement. The simulated basis set included alanine (Ala), ascorbate (Asc), aspartate (Asp), creatine (Cr), gamma‐aminobutyric acid (GABA), glucose (Glc), glutamine (Gln), glutamate (Glu), glycerylphosphorylcholine (GPC), glutathione (GSH), myo‐inositol (Ins), scyllo‐inositol (sIns), lactate (Lac), phosphoethanolamine (PE), phosphocholine (PCho), phosphocreatine (PCr), N‐acetylaspartate (NAA), N‐acetylaspartylglutamate (NAAG), and taurine (Tau). Additionally, the macromolecule (Mac) basis spectrum was derived from measured metabolite‐nulled spectra.

Metabolite concentrations were corrected for T_2_ relaxation of tissue water and cerebrospinal fluid (CSF) content [34, 35]. The CSF fraction of the VOI was computed by segmenting the T_1_‐weighted images over a 3D mask generated using the VOI coordinates saved in the MRS DICOM file. Tissue segmentation was performed in SPM12 [36], whereas a MATLAB (v2019b, MathWorks, Natick, MA, USA) script was used to generate 3D VOI masks. Tissue water fraction values were set to 84% for PFC and 75.8% for HTL [37]. As the apparent T_2_ relaxation rate is slower due to the Carr–Purcell (CP) conditions of semi‐LASER, we used corrected T_2_ relaxation times of water to estimate metabolite concentrations under CP conditions. We assumed that the T_2_ of water under CP conditions is 1.5× longer than the measured free precession T_2_ based on a previous study that compared water T_2_ values measured with LASER and CP‐LASER sequences [38, 39]. Hence, the T_2_ of tissue water was set to 87 ms for PFC [7] and 66 ms for HTL at 7 T. At 3 T, the water T_2_ relaxation time was set to 119 and 99 ms for PFC and HTL, respectively. These values were determined by multiplying the measured free precession T_2_ values for primarily gray and white matter VOI from prior work [40] by 1.5.

Metabolites with mean Cramér–Rao lower bounds (CRLB) of 20% or less, as estimated by LCModel, were included in the analyses. Values of two metabolites were summed if they showed strong negative correlations (*r* < −0.7), which indicate that the fitting of one metabolite influences the other. To identify strongly correlated metabolites, correlation coefficients from all LCModel outputs were averaged for each VOI at each field strength. Additionally, we report the summed values of N‐acetyl‐aspartate and N‐acetyl‐aspartyl‐glutamate (total NAA, tNAA), as commonly reported in the literature, and Glu and Gln (Glu + Gln), and Glc and Taurine (Glc + Tau) if at least one of the individual concentrations did not meet the mean CRLB ≤ 20% criterion.

### 2.4 Quality Control Criteria

Several quality metrics were analyzed: (1) linewidth of water reference peaks (in Hz and in PPM); (2) SNR, outputted by LCModel or manually computed by dividing the NAA peak amplitude by the root mean square error of noise between −1 and −3 ppm (NAApeak/noise); (3) VOI tissue composition for PFC and HTL; and (4) VOI positioning overlap between 3 and 7 T with Dice similarity coefficient (DSC) [41]. The primary quality control criterion for data inclusion was water reference peak linewidth. Water reference linewidths under 13 Hz at 3 T and 19 Hz at 7 T were considered acceptable for analysis [42]. Additionally, any individual transient with a significant phase shift or a residual water peak was excluded before preprocessing and averaging of transients. VOI positioning overlap between sessions was assessed by co‐registering T1‐weighted 3D volumes from the 3 and 7 T scans with both affine and nonlinear registrations using ANTs [43].

### 2.5 Statistical Analysis

Concentrations estimated at 3 T versus 7 T were compared for metabolites with mean CRLB ≤ 20% at either field. tCr ratios were also compared for the same metabolites to mitigate the effect of water scaling. Because data acquisition was interrupted by an upgrade of the 7 T scanner and COVID‐19 pandemic, we compared clinical measures (BMI, A1c, diabetes duration, and blood pressure) between imaging sessions to ensure no significant physiological changes occurred between the 3 and 7 T acquisitions. The order of data acquisition, specifically the number of individuals scanned first at 3 T versus 7 T, was compared to evaluate randomization of the acquisitions. Blood glucose levels during PFC and HTL acquisitions were compared between 3 and 7 T. These were obtained from averaging blood glucose measured every 5 min in the scanner. This timing resulted in 1–2 blood draws during the PFC acquisitions and 4–5 blood draws during the HTL acquisitions.

As the data were collected from the same participants at 3 and 7 T, we performed paired, two‐tailed *t*‐tests for metabolite, data quality, demographics, and clinical outcome (blood glucose during acquisitions, BMI, A1c, diabetes duration, and blood pressure) comparisons. We also computed Pearson correlations between 3 and 7 T concentration estimates. Multiple testing correction was not applied in this study due to its exploratory nature, as the findings are intended to guide future research. Instead, we opted for a more stringent *p* value threshold of *p* < 0.01 than the standard 0.05 for the comparisons.

## 3 Results

### 3.1 Cohort Characteristics, Data Availability and Physiological Status at 3 T vs. 7 T

The cohort included individuals with T1D of ~15 years' duration, normal blood pressure, and well‐controlled glycemia (Table 1). Out of 17 individuals scanned at both 3 and 7 T, one did not complete the 3 T PFC acquisition, another individual did not complete the 3 T HTL acquisition, and three did not complete the 7 T HTL acquisition. As a result, we obtained 16 pairs of PFC data and 13 pairs of HTL data. All available data fulfilled the predefined QC criteria. Nine participants had their 7 T scan first, whereas eight participants had their 3 T scan first, with the average time separation between their two acquisitions being approximately 1 year (median 273 days, interquartile range 385 days). Hemoglobin A1c, BMI, diabetes duration, and blood pressure collected 1 week before imaging and blood glucose levels collected during MRS acquisition showed no significant differences in the physiological status between the 3 and 7 T scans (Table 1).

### 3.2 Analysis of VOI Placements

The analysis of VOI placements showed good within‐person overlap between 3 and 7 T, with an average DSC of ~0.7 for both regions (Table 2). The tissue fraction analysis revealed similar tissue compositions between 3 and 7 T in HTL. However, significant differences were observed in the fractions of gray matter (GM) and CSF in the PFC at 3 T versus 7 T.

**TABLE 2:** Comparison of MRS quality metrics, volumes of interest (VOIs) overlap, and tissue composition at 3 T and 7 T. *P* value was computed with paired, two‐tailed *t*‐test.

Mean (±SD)	HTL		PFC	
	7 T	3 T	7 T	3 T
*N* of transients	256	256	64	64
SNR (PeakNAA/noise)	69 (±11)	38 (±4)	352 (±71)	277 (±38)
Water peak LW (Hz)	9.7 (±2.9)	5.9 (±1)	12.1 (±1.9)	7.1 (±0.8)
Water peak LW (ppm)	0.033 (±0.010)	0.048 (±0.008)	0.041 (±0.007)	0.058 (±0.007)
*p* SNR	0.000		0.004	
*p* water LW Hz	0.000		0.000	
*p* water LW ppm	0.000		0.000	
SNR (LCModel)	24.8 (±3.6)	15.4 (±1.9)	62 (±11.5)	72.1 (±6.3)
FWHM (LCModel, ppm)	0.031 (±0.006)	0.042 (±0.009)	0.026 (±0.007)	0.029 (±0.007)
*p* LCModel SNR	0.000		0.482	
*p* LCModel FWHM	0.001		0.318	
Dice similarity coefficient	0.699 (±0.172)		0.688 (±0.129)	
Tissue frac—GM	53.8 (±9.1)	57.0 (±5.5)	72.2 (±5.8)	67.4 (±4.9)
Tissue frac—WM	26.1 (±10.8)	22.6 (±6.5)	21.3 (±6.3)	20.7 (±5.2)
Tissue frac—CSF	19.0 (±7.3)	19.8 (±7.7)	5.3 (±3.2)	11.4 (±2.3)
*p* GM	0.232		0.002	
*p* WM	0.311		0.698	
*p* CSF	0.428		0.000	

Abbreviations: CSF, cerebrospinal fluid; FWHM, full width at half‐maximum; GM, gray matter; LW, linewidths; ppm, parts per million; SNR, signal‐to‐noise ratio; WM, white matter.

### 3.3 MRS Data Quality and Reliability of Concentration Estimates at 3 and 7 T

**FIGURE 1:**
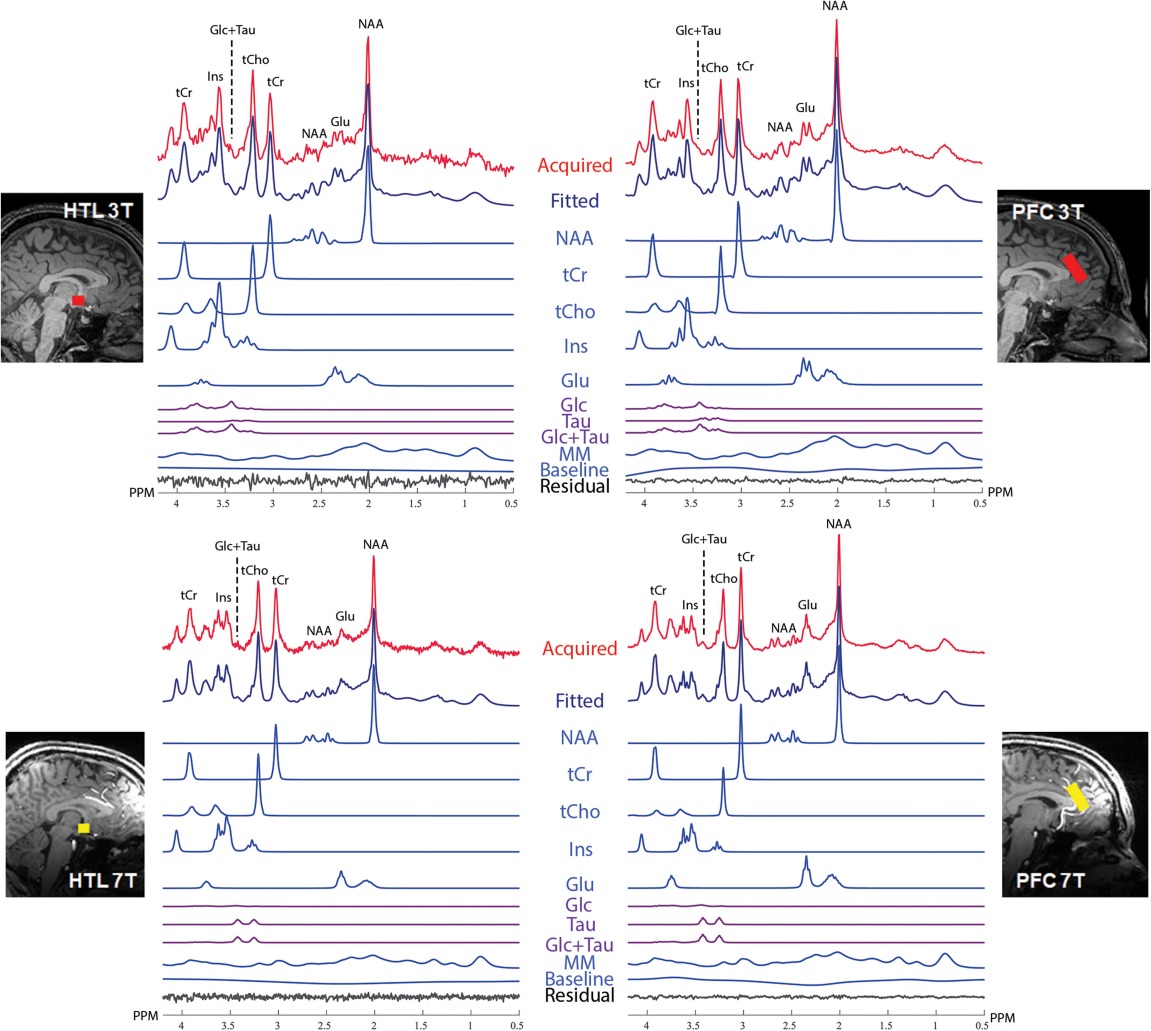
Hypothalamus (HTL, left) and prefrontal cortex (PFC, right) sample spectra and LCModel analysis results from 3 T (top) and 7 T (bottom) are shown together with corresponding voxel placements on T1‐weighted images. Basis spectra of major metabolites, spline baseline, and residuals obtained from LCModel are depicted. Abbreviations are as follows: Glc + Tau, sum of Glc and Tau; Glc, glucose; Glu, glutamate; Ins, inositol; MM, macromolecules; NAA, N‐acetyl‐aspartate; PPM, parts per million; Tau, taurine; tCho, total choline (sum of phosphorylcholine and glycerophosphorylcholine); tCr, total creatine (sum of creatine and phosphocreatine).

The water linewidth was used as a quality control metric during the acquisition because it can be obtained on the scanner. High‐quality spectra were obtained at both field strengths (Table 2 and Figure 1), with the linewidths of water reference peaks well below the expert‐recommended thresholds to exclude data (13 Hz for 3 T and 19 Hz for 7 T) [42]. Other quality control metrics were extracted from the averaged spectra during the analysis and are included per recommendations of the MRS consensus paper on data reporting [44]. 7 T spectra had higher SNR in both VOIs (Table 2 and Table S1). As expected, SNR for HTL was lower than for PFC for both field strengths due to the smaller voxel size of the HTL volume.

**FIGURE 2:**
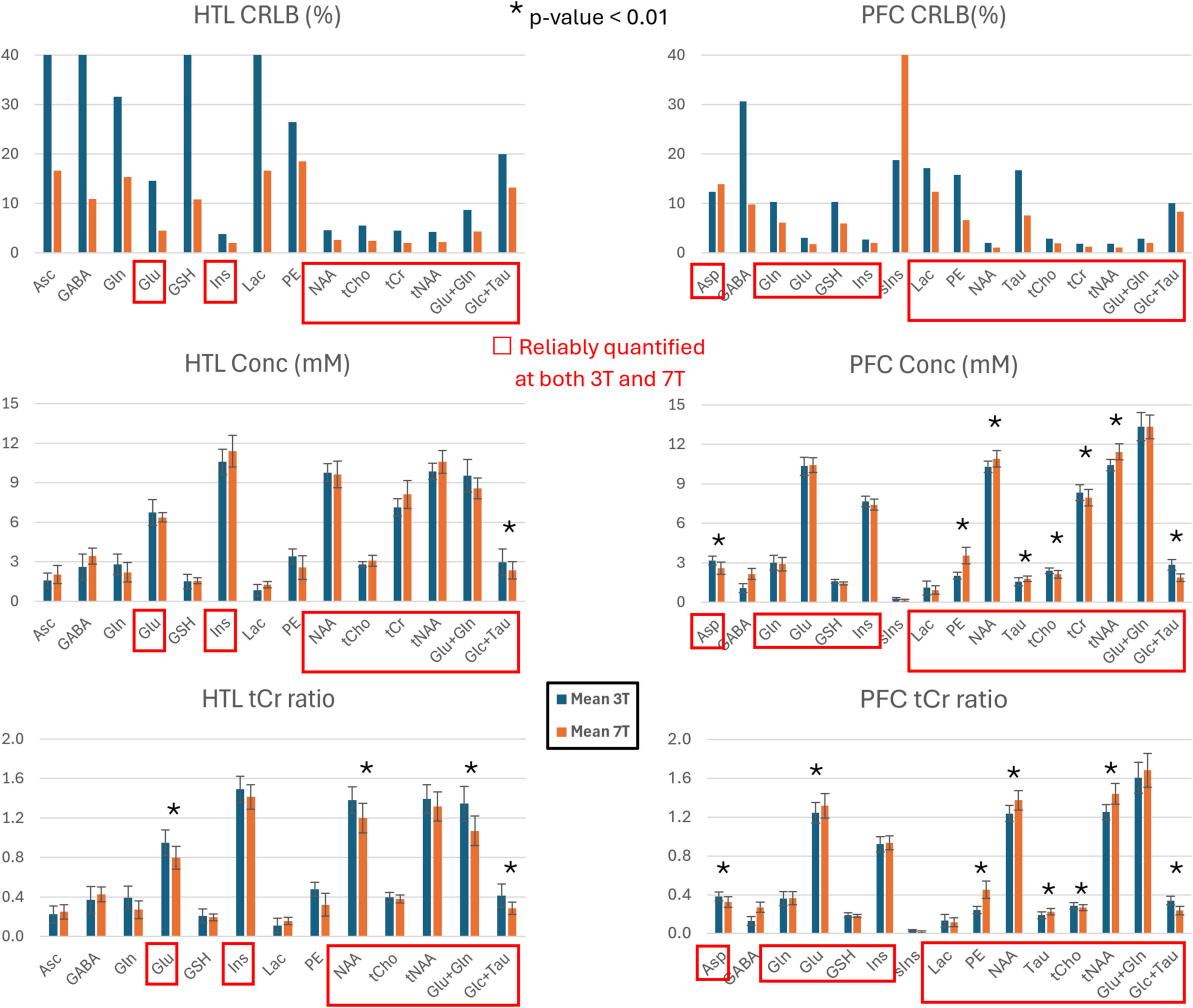
LCModel quantification results of 3 T (blue) and 7 T (orange). Bar plots show mean values and error bars SD. Metabolites with mean CRLB ≤ 20% at either field are shown. Metabolites that were quantified with mean CRLB ≤ 20% at both fields are marked in red boxes. * indicates statistically significant within‐person concentration differences with *p* < 0.01 between 3 and 7 T (paired, two‐tailed *t*‐test). Asc, ascorbate; Asp, aspartate; GABA, gamma‐aminobutyric acid; Glc + Tau, sum of glucose and taurine; Gln, glutamine; Glu + Gln, sum of glutamate and glutamine; Glu, glutamate; Ins, myo‐inositol; Lac, lactate; NAA, N‐acetyl‐aspartate; PE, phosphoethanolamine; Tau, taurine; tCho, total choline (sum of phosphorylcholine and glycerophosphorylcholine); tCr, total creatine (sum of creatine and phosphocreatine); tNAA, total NAA (sum of N‐acetyl‐aspartate and N‐acetyl‐aspartyl‐glutamate).

LCModel concentration estimations yielded 8–15 neurochemicals quantified with mean CRLB ≤ 20% (Figure 2). Metabolite pairs of phosphorylcholine (PCho) and glycerophosphorylcholine (GPC) as well as creatine (Cr) and phosphocreatine (PCr) showed strong negative correlations (*r* < −0.7) at both fields; therefore, their summed values are reported as total choline (tCho) and total creatine (tCr), respectively. A larger number of metabolites were quantified with mean CRLB ≤ 20% at 7 T than 3 T (8 for 3 T vs. 14 for 7 T) in HTL, whereas the number of metabolites reliably quantified for PFC was the same between 3 and 7 T (15 each), with GABA quantified with mean CRLB ≤ 20% only at 7 T and scyllo‐inositol (sIns) only at 3 T (Figure 2 and Table S1).

### 3.4 Metabolite Concentration Estimates at 3 T vs. 7 T

**FIGURE 3:**
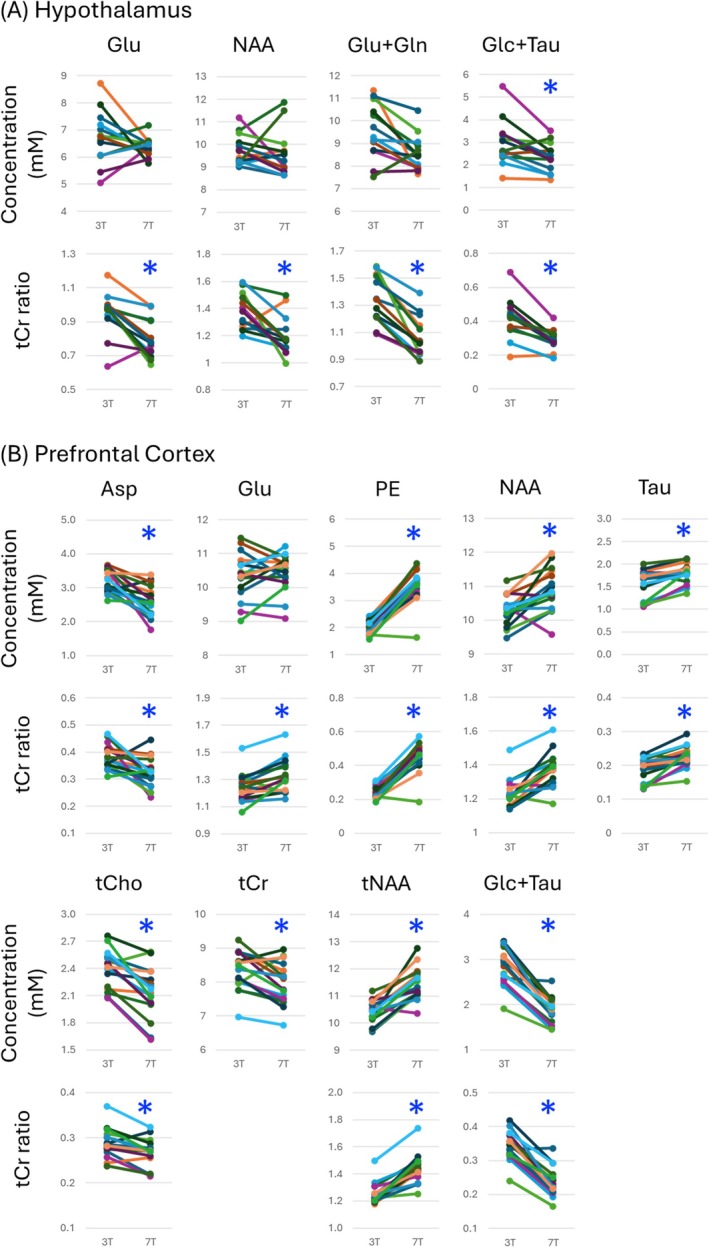
Individual data points at 3 and 7 T for those metabolites quantified reliably (CRLB ≤ 20%) at both fields and displayed statistical significance between fields for (A) hypothalamus and (B) prefrontal cortex. Estimated concentrations (mM) and total creatine (tCr) ratios of metabolites are shown. Stars (*) highlight significant differences between 3 and 7 T with *p* < 0.01 (paired, two‐tailed *t*‐test). Asp, aspartate; Glc + Tau, sum of glucose and taurine; Gln, glutamine; Glu + Gln, sum of glutamate and glutamine; Glu, glutamate; GSH, glutathione; Lac, lactate; mM, millimolar; myo‐Ins, myo‐inositol; NAA, N‐acetyl‐aspartate; PE, phosphoethanolamine; tCho, total choline (sum of phosphorylcholine and glycerophosphorylcholine); tCr, total creatine (sum of creatine and phosphocreatine); tNAA, total NAA (sum of NAA and N‐acetyl‐aspartyl‐glutamate).

For HTL, Glu, Ins, NAA, tCho, tCr, tNAA, Glu + Gln, and Glc + Tau were reliably quantified (CRLB ≤ 20%) at both 3 T and 7 T. In addition, Asc, GABA, Gln, GSH, Lac, and PE in HTL were quantified reliably at 7 T. For PFC, Asp, Gln, Glu, GSH, Ins, Lac, PE, NAA, Tau, tCho, tCr, tNAA, Glu + Gln, and Glc + Tau were reliably quantified at both 3 and 7 T. Whereas most of the mean water‐scaled metabolite levels were comparable at 3 T versus 7 T, a few metabolites such as Glc + Tau (34% higher at 7 T in PFC and 21% in HTL) and phosphoethanolamine (76% higher at 7 T in PFC) displayed notable differences (Figure 2 and Table S2). These differences were systematic across participants and ranged up to 2 mM for Glc + Tau (Figure 3). Quantification with tCr as a reference resulted in a larger number of significant differences between 3 and 7 T due to systematic biases in the tCr concentration between fields (Figures 2 and 3).

### 3.5 Correlations of Metabolite Concentrations Estimated at 3 and 7 T

**FIGURE 4:**
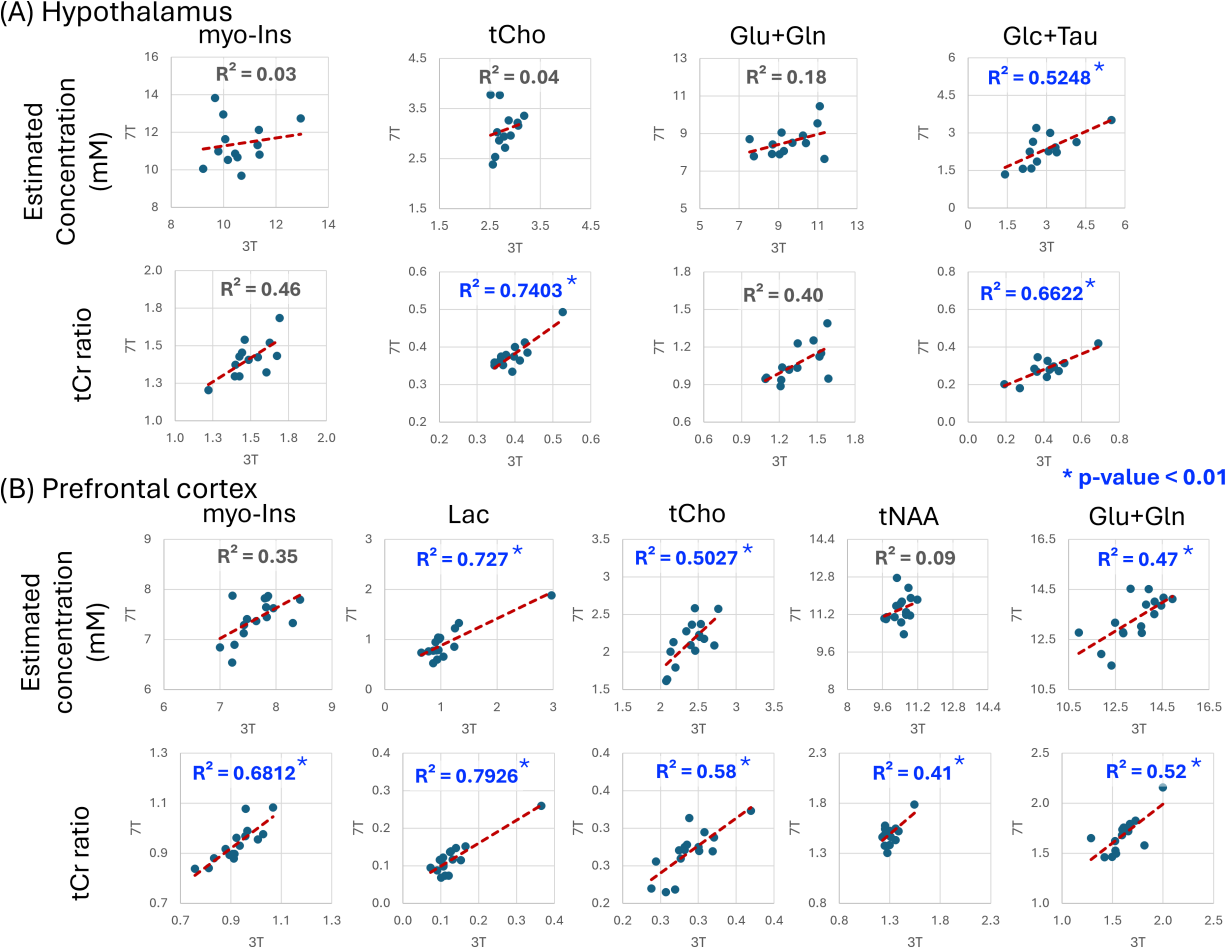
Pearson correlations between 3 and 7 T for (A) hypothalamus and (B) prefrontal cortex for estimated concentrations and total creatine (tCr) ratios of select metabolites are displayed. * and blue color highlight correlations with *p* < 0.01 between 3 and 7 T. Glc + Tau, sum of glucose and taurine; Glu + Gln, sum of glutamate and glutamine; Ins, myo‐Inositol; Lac, lactate; mM, millimolar; PE, phosphoethanolamine; tCho—total choline (sum of phosphorylcholine and glycerophosphorylcholine); tCr, total creatine (sum of creatine and phosphocreatine); tNAA, total NAA (sum of N‐acetyl‐aspartate and N‐acetyl‐aspartyl‐glutamate).

Pearson correlation analysis between 3 and 7 T MRS results showed several metabolites with strong positive correlations (Figure 4 and Table S2). The use of tCr ratios revealed a larger number and stronger correlations between 3 and 7 T data (Figure 4 and Table S3). PFC had a larger number of correlations with *p* < 0.01 between 3 and 7 T compared to HTL, likely due to more reliable metabolite estimation with higher SNR in the larger VOI volume.

## 4 Discussion

This study compared MRS results between 3 and 7 T in a cohort with T1D. The data were collected in the same participants, at the same physiological state, and using nearly identical data acquisition parameters. We report systematic biases in concentration estimates of select metabolites between 3 and 7 T in data collected under controlled glycemic conditions. Quantification with total creatine as reference does not alleviate and even exacerbates the biases. We further report significant correlations between the concentration estimates of select metabolites, such as hypothalamic Glc + Tau, between fields at tightly regulated glycemia, indicating an ability to detect interindividual differences in neurochemical concentrations in this clinical cohort. Of these, the strong correlation of hypothalamic Glc + Tau between fields opens the possibility to detect individualized glucose concentrations in this brain region that participates in the regulation of glucose homeostasis.

### 4.1 Novelty Within Context of Prior Field Comparisons

Previous within‐person field comparisons were only conducted with healthy individuals and often involved developmental hardware (4 T magnet, in‐house coils) [8] or small sample sizes (5–10 participants) [6, 9]. Our study differed from these earlier studies by studying a clinical cohort at two different field strengths, a larger sample size (13–16 pairs of 3 and 7 T data), collection of data from two challenging VOIs (one near the center and another close to the periphery of the brain), use of commercial hardware, advanced pulse sequences with prospective motion correction, and carefully maintained plasma glycemic conditions. The data set was well balanced, with an even split of sex ratio and the number of participants who had their 3 or 7 T scan first.

Our findings are consistent with prior field comparisons in healthy individuals with respect to advantages of UHF for metabolite quantification [6, 7, 8, 9]. Namely, the quantification precision is higher at UHF, as evidenced by lower CRLB for the majority of metabolites. Our study with a clinical cohort also adds to prior findings in healthy individuals: Patients with diabetes have higher between‐subject variability in blood glucose levels, which introduces larger variability in brain glucose levels and in turn may increase the variability in other neurochemicals downstream from glucose metabolism, such as glutamate and glutamine. Therefore, it was important to clamp blood glucose levels at normoglycemia to obtain stable physiology during the MRS acquisitions in this clinical cohort. Furthermore, subtle neurochemical differences, such as lower levels of NAA and glutamate, were demonstrated between patients with T1D and healthy controls and interpreted as neuronal loss or dysfunction [45, 46]. Therefore, our findings support the generalizability of field comparisons in healthy brains.

### 4.2 Consistency of Voxel Placement and MRS Data Quality at 3 T vs. 7 T

No 3–7 T VOI pair had a DSC value of 0, indicating that all VOI pairs had some overlap in voxel coverage. The mean DSC value was similar to the previously reported values for manual VOI placement [47] and consistent between the two regions, suggesting that the discrepancies in VOI placements were within the expected range for manual VOI prescription. Automated VOI prescription was not available for this project because existing methods [47] have not been validated with 7 T structural images that contain large inhomogeneities.

As expected, 7 T data had broader linewidths in Hz than 3 T. Water linewidths of PFC data were broader than those of HTL likely because water linewidths in HTL were influenced by the CSF water signal due to substantial CSF contribution to the HTL VOI. This is evident from the broader LCModel linewidth (FWHM) of HTL, which is the linewidth measured from metabolite peaks.

Higher SNR was observed in PFC compared to HTL by both NAApeak/noise and LCModel estimates, which was expected given the smaller volume of HTL (1.56 mL) compared to PFC (8.64 mL, about 5.5 times larger). Given that the HTL data were collected with four times the number of transients compared to PFC, the ratio of SNR for PFC versus HTL would be about 2.7 times. Hence, the ratio of NAApeak/noise SNR between PFC and HTL of about 5.5 for both 3 and 7 T was due to other factors such as proximity to coil elements and spectral linewidths. The LCModel‐estimated SNR ratios between VOI were more moderate (4 for 3 T and 2.5 for 7 T) because LCModel SNR is computed using the fitted metabolite spectra and the residuals rather than the true noise signal. The metabolite quantification gains of the higher field strength for PFC, the region with very high SNR, were moderate, as evidenced by the same number of metabolites quantified with mean CRLB ≤ 20% for PFC at both field strengths. Beyond this CRLB cutoff, the 7 T PFC data still had better reliability as most metabolites had lower CRLB than 3 T, which was most prominent for weakly represented metabolites like Gln and GSH, as previously observed in healthy volunteers and other VOI [7]. Two metabolites, Asp and sIns, had higher CRLB at 7 T. The lower CRLB at 3 T for Asp is likely due to a simpler spectral pattern of Asp at 3 T, where J‐coupled resonances collapse to a dominant peak at 2.8 ppm (Figure S1). For sIns, which is a singlet, an interaction with another overlapping metabolite that has a more complex spectral pattern at 7 T may cause a higher CRLB at 7 T.

### 4.3 Regional Differences in Metabolite Concentration Estimates

No statistically significant differences were observed in the tissue composition of HTL between 3 and 7 T. However, significant differences in GM and CSF compositions were found in PFC. Because the Dice coefficients were the same for the two VOI, the different tissue composition in the PFC was likely due to image contrast differences or segmentation performance for 3 T versus 7 T data, resulting in overestimation of GM fraction and underestimation of CSF fraction in the cortex at 7 T. Of note, metabolite concentrations largely did not correlate with partial volume of GM or white matter (WM) in either VOI, indicating the limited dynamic range of GM and WM, that is, VOI placement consistency, across participants (Table S4). On the other hand, there were some positive correlations between metabolite levels and CSF fraction at 7 T, indicating a systematic bias in the CSF corrections. As the CSF fraction is an important factor in estimating metabolite concentrations [34, 35], differences in CSF estimates between 3 and 7 T result in systematic differences in the final concentrations.

In HTL, and among metabolites quantified reliably at both fields, only Glc + Tau exhibited a significant difference between 3 and 7 T. In contrast, many metabolites in PFC, notably tCho, tCr, and tNAA, displayed significant differences, likely due to the better quantification precision in this VOI (Figure 2 and Table S2). In addition, these differences were region dependent, for example, mean tCr in HTL was higher at 7 T, whereas tCr in PFC was lower at 7 T. Consequently, using tCr ratios resulted in a larger number of significant differences between 3 and 7 T (Figure 2 and Table S3) because tCr concentration itself was different at the two fields.

Given that blood glucose levels were clamped at euglycemia and not different during the 3 and 7 T scans, the prominent difference in Glc + Tau in both VOIs (~20%–30% lower at 7 T) was not due to glycemic differences. Instead, the spectral patterns of Glc and Tau that are differently influenced by J‐coupling at 3 and 7 T likely resulted in the different estimations of Glc + Tau. Namely, some Glc multiplets collapse into a single peak at 3 T, and the simplified spectral pattern of Glc enables more reliable Glc fitting in the 3 T spectra, thereby capturing more of the Glc signal contribution to the spectrum. Tau, on the other hand, exhibits a simpler spectral pattern at 7 T with prominent peaks at 3.25 and 3.45 ppm (Figure S1). That Glc is more reliably quantified at lower fields was also shown in previous field comparisons, specifically in the occipital cortex at 4 T versus 7 T [8] and in the posterior cingulate and the cerebellum at 3 T versus 7 T [7]. Therefore, whereas other J‐coupled metabolites are more reliably quantified at UHF due to increased spectral dispersion, Glc quantification is an exception to this trend. Another J‐coupled metabolite, GABA, was underestimated at 3 T, even in the high‐quality PFC spectra, demonstrating that it is challenging to reliably quantify with non‐edited MRS at 3 T.

Such differences are not unexpected due to the many factors that influence concentration estimates, including relaxation times that are different across metabolites and between fields, differences in CSF estimates, and the interaction of spectral resolution with spectral patterns that are field dependent due to J‐coupling. Despite these subtle, systematic, and region‐dependent differences, the neurochemical profiles (both concentrations and tCr ratios) and their between‐person variance obtained at the two fields from this diabetic cohort were remarkably similar.

### 4.4 Correlation Analysis

Pearson correlation analysis revealed stronger correlations between 3 and 7 T in PFC than in HTL for both estimated concentrations and tCr ratios because the high SNR in this VOI allowed more reliable detection of metabolite concentrations across participants. For both HTL and PFC, using tCr ratios increased the strength of the associations between 3 and 7 T, further tightening the data by removing potential biases from water scaling. Importantly, metabolites (or their sums) shown in Figure 4 with significant correlations between 3 and 7 T were all quantified with mean CRLB ≤ 20% at both fields, but not all metabolites that were quantified with mean CRLB ≤ 20% at both fields, such as tCr, displayed significant correlations, indicating a smaller dynamic range among individuals. Of particular note was the significant Glc + Tau correlation detected only in the HTL at these tightly regulated euglycemic conditions, indicating that some individuals may have consistently higher or lower hypothalamic Glc + Tau levels than others at the same blood glucose levels. The HTL contains glucose‐sensing neurons and participates in the regulation of the release of counterregulatory hormones such as glucagon, epinephrine, growth hormone, and cortisol to maintain peripheral glucose homeostasis [48, 49]. Hence, consistent hypothalamic Glc + Tau levels across different field strengths demonstrate the utility of MR spectroscopy for measuring hypothalamic Glc + Tau levels in participants, which may be attributable to a range of hypothalamic glucose levels at the same euglycemic blood glucose levels. However, the factors that lead to the observed hypothalamic Glc + Tau range across individuals remain to be determined.

### 4.5 Limitations

Although the differences in the plasma glucose levels between the 3 and 7 T scans were not statistically significant, blood glucose during the HTL measurement tended to be higher at 3 T, and during the PFC measurements tended to be higher at 7 T. We aimed to maintain the participant's plasma glucose level in the 90–100 mg/dL range, with 95 mg/dL as the target, and consistently achieved this in all clamps.

Additionally, we observed significant geometric distortions in 7 T images [50] caused by gradient nonlinearities necessitating nonlinear registration of the brain and VOIs to compute DSCs. This gradient nonlinearity at 7 T could also affect VOI selection, contributing to variations in tissue fractions and quantification results.

Finally, the data used for this study were not prospectively collected for a systematic comparison between 3 and 7 T, leading to minor variations in the pulse sequence and the absence of test–retest measurements. Although the sample size was larger than prior field comparisons, it is still small for definitive conclusions in the correlation analyses. Therefore, a prospective follow‐up study with a larger sample size and test–retest reliability measurements should be undertaken to better understand these data, particularly regarding the relatively small hypothalamic voxel.

## 5 Conclusions

Neurochemical profiles obtained from a cohort with T1D at a target blood glucose of 95 mg/dL using the same MRS protocol at 3 and 7 T are comparable but show systematic biases in select metabolites. Referencing to tCr does not prevent and may even exacerbate the systematic biases. Notably, significant associations between metabolite estimates at the two fields suggest detectable interindividual differences. Additionally, hypothalamic Glc + Tau correlations under tightly regulated euglycemia highlight the potential for individualized glucose concentration detection in this key regulatory brain region.

## Conflicts of Interest

Gülin Öz has consulted for IXICO Technologies Limited, uniQure biopharma B.V., VICO Therapeutics, Servier, Sanofi, and UCB Biopharma SRL/Lacerta Therapeutics Inc., serves on the Scientific Advisory Board of BrainSpec Inc., and received research support from Biogen.

## Supporting information


**Table S1.** Cramér–Rao Lower Bounds results.
**Table S2.** Metabolite concentration results.
**Table S3.** Metabolite tCr Ratio results.
**Table S4.** Correlations between VOI tissue fraction (white and gray matter) to metabolite concentration and tCr ratio.
**Figure S1.** Comparison of basis spectra.

## Data Availability

The data that support the findings of this study are available on request from the corresponding author. The data are not publicly available due to privacy or ethical restrictions.
