# Supplementary material for: Neurochemical Profiles of Prefrontal Cortex and Hypothalamus at 3 and 7 T During Controlled Euglycemia: Evaluation in a Cohort With Type 1 Diabetes
Source: NMR Biomed. 2025 Jul 27;38(9):e70108. doi: 10.1002/nbm.70108 (PMC12301584; doi:10.1002/nbm.70108)
Supplement: Supplementary file 1 — Table S1. Cramér–Rao Lower Bounds results. Table S2. Metabolite concentration results. Table S3. Metabolite tCr Ratio results. Table S4. Correlations between VOI tissue fraction (white and gray matter) to metabolite concentration and tCr ratio. Figure S1. Comparison of basis spectra. [file NBM-38-e70108-s001.docx]

**Supplementary Table S1 – Cramér-Rao Lower Bounds results**

**Supplementary Table S2 – Metabolite concentration results**

**Supplementary Table S3 – Metabolite tCr Ratio results**

**Supplementary Table S4 – Correlations between VOI tissue fraction (white and gray matter) to metabolite concentration and tCr ratio**

**ST4.1** Metabolite concentrations

In hypothalamus, statistically significant correlations (p<0.01, paired, 2-tailed t-test) were seen between 7T myo-Inositol (Ins) and white matter (WM), 7T Ins and cerebrospinal fluid (CSF), 7T sum of choline and phosphocholine (total Choline, tCho) and WM, and 7T Glc+Tau and CSF. In prefrontal cortex, significant correlations were observed between 3T sum of creatine and phosphocreatine (total Creatine, tCr) and CSF, 7T glutathione (GSH) and CSF, 7T sum of N-Acetyl-Aspartate and N-Acetyl-Aspartyl-Glutamate (total NAA, tNAA) and CSF. Note that multiple testing correction was not applied in this analysis due to its exploratory nature.

**ST4.2** Metabolite tCr ratio

No statistically significant correlations were found between the tissue fraction and metabolite tCr ratio in hypothalamus. In prefrontal cortex, a significant correlation was observed between 3T N-Acetyl-Aspartate (NAA) and CSF. Note that multiple testing correction was also not applied in this analysis due to its exploratory nature.

**Supplementary Figure S1 – Comparison of basis spectra.**

**SF1.1** Basis spectra of Glucose and Taurine

**SF1.1.1** 7T LC Model basis spectra of Glucose, Taurine and summed spectra of Glucose and Taurine
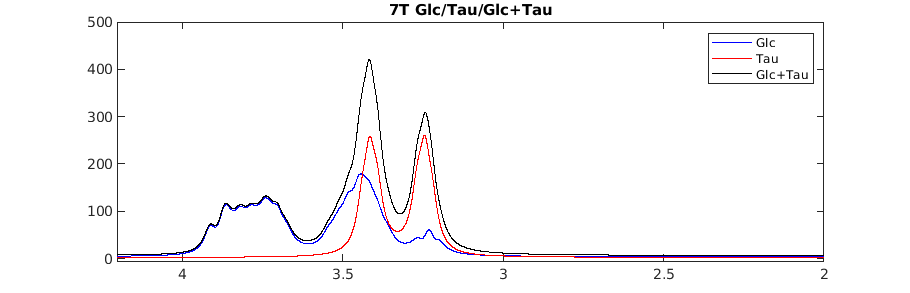


7T (TE_1_/TE_2_/TE_3_ = 7 / 10 / 9 ms ); 7T TE_total_ = 26 ms

**SF1.1.2** 3T LC Model basis spectra of Glucose, Taurine and summed spectra of Glucose and Taurine
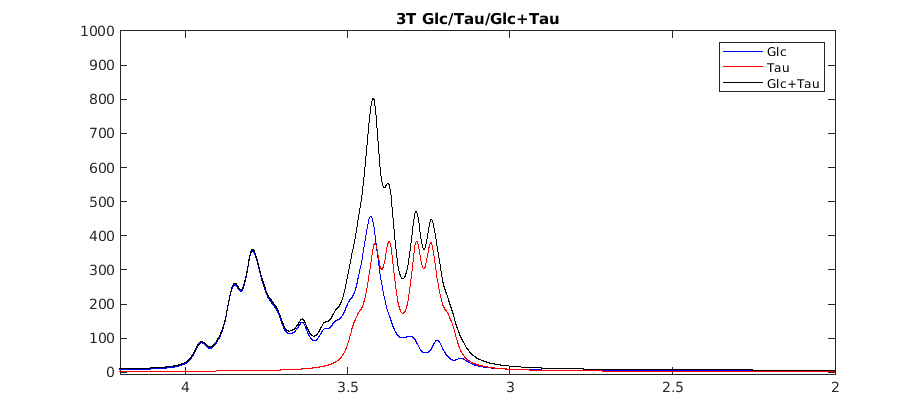


3T (TE_1_/TE_2_/TE_3_ = 8 / 11 / 9 ms ); 3T TE_total_ = 28 ms

**SF1.1.3** 3T vs 7T comparison of basis Glucose spectra
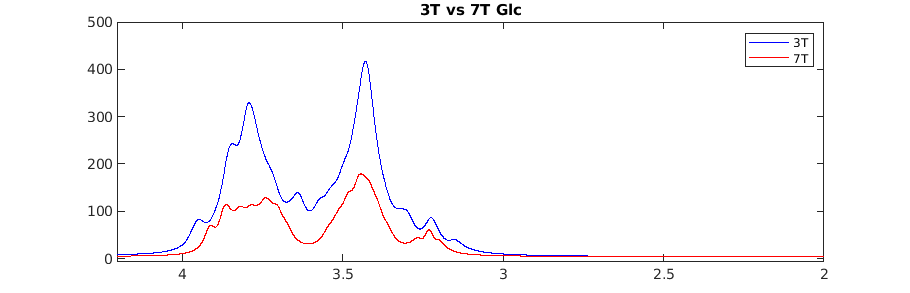


3T and 7T basis spectra of Glucose (Glc) are displayed. The spectral linewidths were broadened to 7Hz and 12 Hz respectively to match the mean linewidths of PFC data. For 3T spectra, two prominent spectral peaks are visible at 3.4 and 3.75 ppm. On 7T spectra a complex spectral pattern results from J-coupling for the same peaks.

**SF1.1.4** 3T vs 7T comparison of basis Taurine spectra
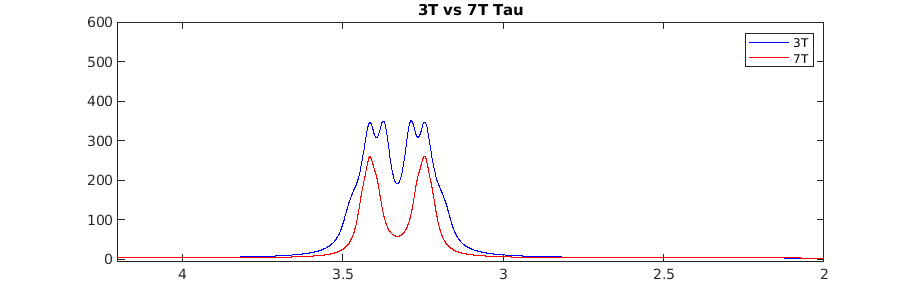


3T and 7T basis spectra of Taurine (Tau) are displayed. The spectral linewidths were broadened to 7Hz and 12 Hz respectively to match the mean linewidths of PFC data.

**SF1.2** Basis spectra of Aspartate

**SF1.2.1** 3T vs 7T comparison of basis Aspartate spectra
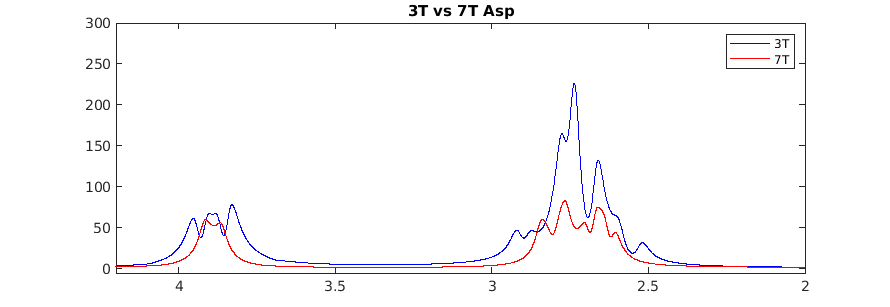


Our 3T and 7T basis spectra of Aspartate (Asp) is displayed. Note that the spectral linewidths have been broadened to 7Hz and 12 Hz respectively to match the mean linewidths for PFC data. For 3T spectra, a prominent spectral peak is visible at 2.8 ppm, which is not the case on the 7T spectra.
